# Developement of Teeth in Aged Persons,—or, Third Dentition

**Published:** 1840-07

**Authors:** Harvy Burdell


### EXTRACTS

*From professor* Blandin's "*Anatomie du Systime Dentaire,*"—
*Translated from the French, with Notes,*

[ BY HARVY BURDELL, M. D. ]

### DEVELOPEMENT OF TEETH IN AGED PERSONS,—OR,

### Third Dentition.

Two dentitions on the the developement of the infant and adult teeth, are all that persons require in ordinary cases during life, and if it were not owing to the many deleterious agents often operating upon them, either directly or through the medium of the general organization, the permanent or adult teeth would undoubtedly remain in the mouth, healthy and unimpaired throughout the whole period of our existence. We also observe that two sets of teeth characterize the human species, and that any variations from these physical laws are extremely rare ; yet when cases of a third dental formation happen, we ought to consider such anomalies as sports of nature, and as indicative of the regenerative powers, she possesses although in this respect but seldom exercised. *

Joubert reported a case in which a lady of nobility lost all of her teeth, and at the age of seventy, twenty new teeth grew from her jaws.

Seunert records a similar case, in which a lady of Silesia of about the same age, had twenty teeth renewed, and the symptoms attending this dentition, he says, were similar to those attending the dentition of infants.

Eustachi saw a young man who lost his incisors at the age of twenty, and during the same year, four new teeth appeared in the spaces of the former.

Duffay, a celebrated physician of the East Indies, saw a man who had two incisors and two canines produced at the age of eighty.

Gehler witnessed a case in which an eye tooth had been renewed three times. Hunter relates a case of the appearance of teeth at the age of seventy years.† In a case which came under my own notice, I found in the jaw of an adult just below the first bicuspid, a new tooth, the crown of which had not yet perfectly formed. * * *

The views and opinions entertained by authors, relative to a third dentition, or of the re-production of teeth in old age, are at variance with each other. Some of them contend that the developement of teeth, late in life, is simply owing to the circumstance, that the first or second set had only been retarded, and consequently appeared at a more advanced age ; while others deny this to be the case, and offer different theories in proof of their doctrines. Among the writings of dental authors, there appears to be much exaggeration relative to the growth of a third set of teeth, and consequently, erroneous positions have been assumed. In the case witnessed by Gehler, for instance, was it possible for this tooth to

---

* The late and much lamented Dr. John Mason Good, whose name is mentioned by learned men with the deepest veneration for his immortal memory, thus writes : "sometimes," says he, "though rarely, we meet with playful attempts on the part of nature to re-produce teeth at a very late period of life, and after the permanent teeth have been lost by accident or decay. This most commonly takes place between the sixty-third and the eighty-first years, or the interval which fills up the two grand climacteric years of the Greek Physiologists ; *at which period the constitution appears occasionally to make an effort to repair other defects than lost teeth.*" Many other learned physiologists coincide with the opinions of Dr. Good.—H. B.

† Only a single instance, in which the alveolar sockets were renewed, came under the notice of that celebrated surgeon, John Hunter.

Mr. E. Parmly, of this city, in his notes to Brown's Dentologia, records the case of an adult who never had had any teeth, yet the alveolar processes were developed so as to fill up the vacancy in the mouth, which would have manifested itself had the teeth been lost by disease or extracted.

Dr. D. Rosseter of this city, also met with a case in his own practice, similar to that mentioned by Mr. P. Dr. Rosseter states that the alveoli and gums were developed in this case so as to be used as teeth, the surfaces coming in contact during mastication, being sharp like the crowns of the teeth.—H. B.

have belonged to the second set, when it had been renewed three times ? On the contrary, is it probable that the rudiments of three teeth of the same class, could have been originally formed in the jaw, in opposition to what has already been said upon the subject ? * * *

Why have so many writers opposed the doctrine of a third dental production ? Is it not quite as difficult to enter upon a full and philosophical explanation of the more important anomalies met with in the general and comparative anatomy of man, and also of animals, than it is in those which happen in the developement of teeth ? Certainly ! without doubt ! * * *

The best authenticated cases upon record, are those in which only one or two teeth have been renewed.* Hunter says, that it sometimes happens, that a third set of teeth appear in the jaws of very old people ; but in reference to such instances, he very justly observes, that when teeth appear so late in life, they cause much inconvenience, and are more *hurtful* than *useful*, as they usually appear only in one jaw, and consequently, are apt to wound the gums of the other when coming in contact, and in such cases, it is necessary to extract them.

* I have witnessed one case of this kind, in my own practice. In this instance, the patient was aged about seventy years, and he called "to have the last tooth in his head extracted," as he remarked. He said it was very troublesome, but not in the least decayed. The tooth was very white, and appeared like an infant lateral incisor. I extracted it with slight effort, and on splitting through from the end of its root to the extremity of the crown, found it perfectly solid, there not being any nerve or canal in its centre.—H. B.

